# Advance care planning and quality of life: A qualitative interview study in people with young-onset dementia and their family caregivers

**DOI:** 10.1177/02692163251324796

**Published:** 2025-03-15

**Authors:** Jasper Maters, Marieke Perry, Ton de Wit, Raymond T.C.M. Koopmans, Marjolein E. de Vugt, Christian Bakker, Jenny T. van der Steen

**Affiliations:** 1 Department of Primary and Community Care, Radboud university medical center, Nijmegen, the Netherlands; 2 Radboudumc Alzheimer Center, Nijmegen, the Netherlands; 3 Department of Geriatrics, Radboud university medical center, Nijmegen, the Netherlands; 4 Mijzo, Center for Elderly Care, Waalwijk, the Netherlands; 5 Joachim en Anna, Center for Specialized Geriatric Care, Nijmegen, the Netherlands; 6 Alzheimer Center Limburg, Mental Health and Neuroscience research institute, Maastricht University Medical Center, Maastricht, the Netherlands; 7 Groenhuysen, Center for Geriatric Care, Roosendaal, the Netherlands; 8 Department of Public Health and Primary Care, Leiden University Medical Center, Leiden, the Netherlands; 9 Cicely Saunders Institute, King’s College London, London, UK

**Keywords:** Advance care planning, palliative care, young-onset dementia, qualitative research, interview

## Abstract

**Background:**

The importance of palliative care and advance care planning in dementia is increasingly recognized. However, little is known about the distinct needs and preferences of people with young-onset dementia.

**Aim:**

To explore how people with young-onset dementia and their family caregivers experience quality of life, and how these experiences shape their views on the future, palliative care needs, and advance care planning.

**Design:**

A qualitative interview study, using inductive thematic analysis.

**Setting/participants:**

Ten community-dwelling people with young-onset dementia and their family caregivers from the Netherlands.

**Results:**

Four themes were found: sense of self, connection to others, acceptance versus resistance in the face of adversity, and orientation toward the future versus focus on the present. The first two themes represent the main aspects of quality of life. The third theme covers acceptance versus resistance in coping with the disease, support, and the future. The expectations of a decrease in quality of life could adversely affect the attitude toward the future and thus reduce the willingness to engage in advance care planning, which relates to the final theme. Quality of life explicitly influenced treatment decisions for those who engaged in advance care planning.

**Conclusions:**

The perception of young-onset dementia and its impact on quality of life varies among people with young-onset dementia and their family caregivers, but common values include a sense of self and connection to others. Advance care planning should be introduced as a way to protect these values and align them with palliative care goals.

**What is already known about the topic?**

- Young-onset dementia impacts quality of life and requires an age-appropriate approach to care that is tailored to individual needs and is family oriented.
- Advance care planning is considered important in young-onset dementia.
- A relational, flexible, and holistic approach has been recommended when initiating advance care planning.

**What does this paper add?**

- This study on young-onset dementia identified future care preferences consistent with a palliative care approach, emphasizing palliative care goals and prioritizing quality of life in decision-making.
- Differences were found between perspectives of people with young-onset dementia and those of their family caregivers: caregivers displayed less positive attitudes toward the disease and tended to postpone decision-making more often.
- Negative perceptions of future quality of life can prevent people from engaging in advance care planning.

**Implications for practice, theory or policy**

- Healthcare professionals should initiate advance care planning discussions with individuals with young-onset dementia and their family caregivers, allowing each to express their distinct perspectives before aligning them.
- The topics of preserving a sense of self and connection to others can help initiate discussions about palliative care.
- Understanding individuals’ views on their future quality of life can remove barriers to advance care planning, empowering patients to make decisions about their care.

## Introduction

Around 4 million individuals worldwide are living with young-onset dementia, with symptom presentation before the age of 65.^1,2^ Young-onset dementia is clinically different from late-onset dementia. Differences include a different distribution of etiologies, fewer comorbidities, and longer survival duration.^3–5^ People with young-onset dementia have specific care needs as a result of their age, life stage and family life stage.^6^ Previous research stresses the need for an age-appropriate approach to care that is tailored to individual needs and family oriented.^7,8^

In light of young-onset dementia’s profound impact on quality of life, people could benefit from a palliative care approach which aims to improve quality of life^9–11^ Recently, a dementia-specific palliative care goals model has been developed with quality of life as main focus.^10^

An important domain of palliative care revolves around person-centered care, communication and shared decision making that includes people with dementia and family.^12^ For this, we need to understand the perspectives of people with young-onset dementia and their family caregivers on future care. Their views remain understudied, even though they are expected to differ from those of people with late-onset dementia.^13^

To ensure that palliative care is tailored to individual needs of people with late- and young-onset dementia, advance care planning is essential.^14^ Advance care planning aims to align treatment and care decisions with personal values, thereby promoting a person-centered approach.^15,16^ It is defined as a process of ongoing communication about future care and treatment preferences, values and goals with the person with dementia, family, and the healthcare team.^17^ An international Delphi study identified capacity, family involvement, and engagement and communication as three key issues that differ for advance care planning in the case of dementia.^17^ It emphasized involving the person with dementia as long as possible, recognizing their declining decision-making capacity, which may eventually necessitate shifting engagement and communication from the person to their family.

Physicians acknowledge the added value of advance care planning for people with young-onset dementia and their family caregivers.^18^ However, they encounter various challenges regarding engaging in the process. People with young-onset dementia and caregivers highlighted a gap in knowledge, information, and age-appropriate care as barriers.^19^ A relational, flexible, and holistic approach when initiating advance care planning is recommended.^20^

So far, the topics of quality of life and advance care planning have been studied separately in young-onset dementia. Little is known about the role of quality of life in advance care planning. Therefore, the research question of this study is: how do people with young-onset dementia and their family caregivers experience quality of life, and how these experiences shape their views on the future, palliative care needs, and advance care planning?

## Methods

### Study design

We adopted a qualitative study design, conducting semi-structured face-to-face in-depth interviews.^21^ COREQ guidelines were followed for reporting.^22^

### Population

We used purposeful sampling (Table 1) of dyads of community-dwelling people with young-onset dementia and their family caregivers. Our aim was to ensure diversity in terms of sex, level of education, type of dementia, ethnic background, and dyadic relationship. We estimated that interviewing ten dyads would provide sufficient diversity to support a meaningful analysis.^23^

**Table 1.** Eligibility criteria.

Person with young-onset dementia	Family caregiver	
Inclusion criteria		
• Willing and able to reflect on situation, illness, care preferences, and any future developments • Capable of providing informed consent		
• Onset of first dementia symptoms prior to age 65 (diagnosis can be after age 65) • Mild to moderate dementia based on the case managers’ clinical judgement using the adapted Dementia Severity Rating Scale • Living at home		• Being the primary caregiver for the person with dementia, living or not living with the person with dementia
Exclusion criteria		
• Alcohol-related dementia, dementia due to Down syndrome, Huntington's disease, and acquired brain injury • Severe aphasia or language impairment		• Barely having contact with person with dementia • Severe aphasia or language impairment

### Setting

Participants lived across the Netherlands, including both rural and urban areas. The Dutch healthcare system provides accessible primary care, including palliative care, with general practitioners well-positioned to facilitate advance care planning.^24^ However, research indicates that advance care planning conservations are still rarely recorded for people with dementia.^25^ While euthanasia is legally permitted in the Netherlands, it remains rare in case of dementia.^24,26^

### Recruitment

Dementia case managers were approached through information leaflets distributed by the national Young-onset Dementia Knowledge Center.^7^ They identified potential participants and evaluated their cognitive abilities by using the Dementia Severity Rating Scale, an 11-item informant-based questionnaire assessing dementia severity.^27^ To select participants capable of being interviewed and to consent to participate, they were advised to use cut-off values for three items: memory (2), speech and language (3), and ability to make decisions (2). The case managers asked eligible participants for permission to be contacted by the researcher (JM or TdW, males) by phone. Both worked as physicians in dementia care and were trained in qualitative research methods. Upon agreement, the researcher sent the patient information letter and scheduled the interview at the participant’s place of choice.

### Data collection

Data collection started late 2019, but was discontinued in early 2020 due to the COVID-19 pandemic. It resumed in October 2021 and was concluded in February 2022.

Prior to each interview, participants completed a questionnaire on demographics and the date of diagnosis. Two interviewers simultaneously interviewed the person with dementia and the caregiver in separate rooms. In total, five interviewers (JM, TdW, JvdS, CB, and CO-C) were involved.

The interview started with a question about an item with special meaning which the participant had selected in advance (object elicitation).^28^ The flexibly used topic guide (Supplemental material S1) covered quality of life, the impact of young-onset dementia, (receiving) care, the future, and advance care planning. It included three changes made after conducting five dyadic interviews. The researchers wrote brief field notes to document their observations. All interviews were audiotaped and transcribed verbatim. The first author performed member checks by phone with participants who were interviewed after the study's relaunch. After completion of the analysis, participants were informed about the results via a newsletter.

### Data analysis

We performed inductive thematic analysis following the approach of Braun and Clarke.^29,30^ This method was chosen for its ability to foster a deep understanding of the participants’ experiences.

All transcripts were checked for correctness and anonymized. JM reviewed the transcripts line-by-line and assigned initial codes using ATLAS.ti (version 23.1.1). Ten transcripts were independently reviewed and coded by JM and a second researcher (TdW, JvdS, and MP), for purpose of comparing standpoints and improving reflexivity. Subsequently, the initial codes were refined and grouped into code groups, subthemes, and themes through a collaborative effort (JM, JvdS, and MP) in a series of meetings. The researchers frequently returned to the text fragments to check whether codes captured their meaning. JvdS and MP have extensive experience in qualitative research on palliative care and dementia. Data abstraction was a flexible, iterative, and reflexive process. Finally, the findings were reviewed by RK, CB, and MvdV, all experienced researchers with a special interest in young-onset dementia.

### Ethics

The study (ICTRP ID NL-OMON23226) was conducted according to the principles of the Declaration of Helsinki (version 13) and ICMJE Recommendations. The Research Ethics Committee (CMO Regio Arnhem-Nijmegen) approved this study (number 2019-5445) on 14-08-2019. The study fell under the Medical Research Involving Human Subjects Act (WMO) as the medical ethic committee considered the sensitive nature of the interview topics and the interviewees being persons with dementia. Every participant provided written informed consent prior to the interview. The interview guide included instructions for the interviewers to pause the interview in case of visible stress. Participants were offered the opportunity to contact an independent physician, as stated in the information letter.

## Results

In total, 13 dyads were approached. One dyad was excluded because of severe language impairment. Two dyads withdrew due to COVID-restrictions. Interviews with the remaining ten dyads lasted 67 min on average. All took place at the participants’ home.

The age of participants with dementia ranged from 53 to 71 years (Table 2). Eight family caregivers were spouses. All participants had a white Dutch background. Alzheimer’s dementia was most common (*N* = 7). Seven dyads had an advance directive.

**Table 2.** Characteristics of dyads (*N* = 10).

Variable	People with young-onset dementia (P01–P10)	Family caregivers (C01–C10)
Gender		
Male	7	2
Female	3	8
Age (years)		
Range	53–71	52–69
Mean	60.2	59.5
Education		
Secondary	1	4
Vocational	6	2
University	3	4
Dyadic relationship		
Spouses	8	
Unmarried couple	1	
Friends	1	
Dementia type		
Alzheimer’s dementia	6	
Frontotemporal dementia	1	
Lewy body dementia	1	
Posterior cortical atrophy	1	
Time since diagnosis		
Range	7 months–6 years and 4 months	
Mean	1.8 years	
Advance directive		
Power of attorney	6 and 2 being worked on	
Living will	4 and 2 being worked on	
Euthanasia statement	2 and 2 being worked on	
No	2	

Thematic analysis yielded four themes (Table 3): (1) sense of self; (2) connection to others; (3) acceptance versus resistance in the face of adversity; and (4) orientation toward the future versus focus on the present. Sense of self and connection to others were identified as the core values of quality of life. Acceptance versus resistance in the face of adversity related to how participants coped with their situation. The final theme revolved around how they approached their future, proactively versus reactively.

**Table 3.** Themes, subthemes, and code groups.

Theme	Subtheme	Code group
Sense of self	Autonomy	Importance of freedom of movement
		Wish not to be dependent on others
		Wishes of person with dementia must be central
	Importance of own identity	Importance of having skills
		Ability to enjoy one’s own activities
		Individual perception of quality of life
		Tendency to hold on to the past
		Importance of a familiar environment
	Feeling useful	Importance of activities on a daily basis
		Importance of meaning something to others
		Importance of meaningful activities
Connection to others	Importance of being in contact with others	Importance of contact with family members
		Value of contact with fellow patients
		Engagement in social activities
		Maintaining social relationships
		Familiarity important in interactions
	Thinking of well-being of others	Wish to not burden others too much
		Well-being of family perceived as important
	Negative impact of dementia on relationships	Limitations in social interaction
		Feeling of losing the person with dementia
		Losing reciprocity in relationship
		Negative perceptions of others about dementia
Acceptance versus resistance in the face of adversity	Adjustment to limitations	Need for others to adapt to the person with dementia
		Taking impairments into account by person with dementia themselves
		Tendency not to dwell on mistakes by dyad
	Acceptance of current situation	Acceptance of current situation
	Challenges regarding coping	Wish to get answers to existential questions
		Perception that dementia has a profound impact on life
		Difficulty accepting diagnosis and limitations
		Not wanting to leave a negative impression on others
		Not having had the time to prepare for living with dementia
		Not wanting to discuss dementia with others
	Openness to help of others	Agreement with current support
		Expectation of growing role of partner in future in case of decline
		Belief that others can help with advance care planning
		Importance of being open about the situation
		Positive perception of current support
	Negative outlook on the future	Fear of death
		Fear that dementia will devalue patient as a person
		Negative view of decline
	Negative view of support	Belief that others cannot really help
		Negative view of living in a nursing home
		Perception that support does not meet personal needs
	Perception that decline is inevitable	Awareness of dementia deterioration
		Perception that there is no treatment
		Tendency to think about the end of life
	Positive attitude toward the situation	Dementia has also had positive implications
		Experience that there is quality of life in spite of dementia
		Hopeful view toward the future
		Focus on what is still possible
Orientation toward the future versus focus on the present	Current wishes with regard to the future	Euthanasia is not an option
		Euthanasia is a possibility in case of decline
		Quality of life important when making treatment decisions
		Wish to avoid suffering in final phase
		Extension of life is not always desirable
		Not wanting to live in a nursing home
	Engagement with the present rather than the future	Adopting a day-to-day attitude
		Tendency to not think of the future
		Discussing the future is confronting
	Preparing for the future is pointless	Mental incapacity will hinder the execution of made plans
		Uncertainty what the future will hold
		Wishes may change over time
	Tendency to postpone decisions	Wish to decide at the moment a decision is required
		Tendency to leave the situation unchanged until it becomes untenable
		Trust that caregiver will make decisions in the future
	Wish for certainty toward the future	Importance of good information about dementia
		Thinking about future scenarios
		Peace of mind from making and documenting arrangements
		Wish to document matters while this is still possible
		Preparations for possible future scenarios

### Sense of self

Quality of life stemmed from a sense of self: being an autonomous individual who values own identity and wants to feel useful. Own identity included appreciation of personal activities, surroundings, past achievements and habits. Participants considered retaining abilities in spite of cognitive decline important. Most dyads deemed quality of life to be based on individual values to be judged by the person with dementia themselves. *CO6: (about difference of opinion on quality of life between him and partner) “Yes I think it’s very relative. Everyone should judge for themselves I think.”*

Regarding autonomy, there was a strong desire not to be dependent upon others, especially for personal care. The importance of moving freely outside the home was emphasized. *C03: “And are there any other things that make his life worthwhile? That he considers important in life? Yes, his independence and also driving, you know, that, well, that’s also a dilemma. He still drives a car now.”* When medical decisions needed to be made, the dyads stated that ultimately the person with dementia should decide. Many caregivers stressed the importance of self-determination of the person with dementia even if their opinion was different. *C04: “Obviously that’s what’s important, because he doesn’t want what I’d want for myself or how I would want it for him. (. . .) in the end it’s about what he wants of course.”*

Compared to their caregivers, persons with dementia more frequently mentioned the importance of feeling useful. They derived fulfillment from meaningful activities and contributing to the well-being of others, for example volunteer work. Further, they preferred a daily schedule with regular activities. *P10: “Just having something to do (. . .) I want to continue the activities I do now, because if I sit in my chair all day, that won’t work.”*

### Connection to others

Connection to others was essential for quality of life as it contributed to a sense of belonging despite challenges posed by dementia. The participants with dementia wanted to maintain relationships and engage in social activities as long as possible. Almost all stressed the central role of family in their life. C02: *“I remember him saying, ‘if I don’t recognize my children and you anymore, then I don’t know how much quality there is,’ because the fact that you can share things and have a history together, the family that we started together is the foundation of what became our life, and the moment that foundation is gone, then the rest is unsteady too.”* Contact with other persons with dementia was considered valuable as it allowed for sharing mutual experiences.

Dyads considered the well-being of others. Not wanting to put too much strain on others could keep them from asking for support. The well-being of family members, in particular their spouse, was perceived as important by participants with dementia. *P02: “I’m not going to tell him (husband) ‘You have to keep taking care of me’ if it gets too hard. No, I don’t want that, I really don’t. I’d hope he will enjoy life and later maybe meet someone else or whatever. No, I’m not like that, I’d just let it go. No I love him too much for that.”* Many wanted their spouses to have their own lives besides caregiving.

Caregivers felt they were gradually losing their partner and experienced a growing lack of reciprocity in the relationship. Cognitive decline made social interactions more difficult and tiresome. Participants experienced stigma of dementia. *P09: (the interviewee was looking for volunteer work) “But I was also disappointed a few times when the message was: you’re no use to us.”*

### Acceptance versus resistance in the face of adversity

The interviews revealed varying attitudes toward the disease, support, and the future. Coping styles varied greatly among participants, ranging from acceptance to resistance. Some of the participants had come to terms with their diagnosis which helped them find a sense of peace. *P07: “Well, you can’t really get angry about that it happens to you. Nothing you can do about it. I'm at peace with that.”*

By contrast, others found it hard to accept their situation. The loss of control elicited various negative emotions. For some, dementia had a profound impact on their quality of life. *C02: “I think for her there is only one quality of better life: that the disease can be cured. So that's what she tells me every morning. It’s the first thing on her mind every day.”* Many interviewees expressed a sense of unpreparedness for living with dementia, as their lives had suddenly been turned upside down, leaving them with existential questions. *P02: “Then I think: why did this happen to me? But you don’t get an answer to that question either.”* Some preferred not to talk about their disease with others including their partner, and did not want to leave a negative impression on others, including healthcare professionals.

Participants with dementia displayed a positive attitude more frequently than their caregivers, mentioning benefits like having more spare time due to their dementia. Some still experienced enough quality of life despite their dementia, focusing on their remaining skills and enjoying life’s small pleasures. *P10: “I try to enjoy it all as long as possible with what I can still do.”* Some participants maintained an optimistic view toward the future, hoping for slow dementia progression.

As a way of coping, people with dementia consciously took into account their impairments. For example, they did not plan too many activities in a day. Some dyads decided not to dwell on mistakes but rather move on or overlook odd behaviors. *C09: “We almost had a fire once, because he had gone cycling while the soup was cooking on the stove. (. . .) We said: was that important? No it wasn’t important, it’s behind us, done.”*

Regarding support, multiple dyads expressed satisfaction with current help, both professional and informal. Positive experiences made participants with dementia more open to future support, including future planning. Conversely, support was perceived negatively when it did not address the personal needs. Many participants had a negative view of nursing homes, expecting impersonal and low-quality care. Some believed that others could not actually help with their situation. *C03: (talking about the neurologist) “Look, the last conversation we had there went more or less like, if there’s anything we can ever do for you, you’re more than welcome, but for now we’re done. And of course they are because they can’t do anything else for him because there is nothing else.”* A few dyads felt that they essentially had to deal with dementia themselves. *C08: “No, I have thought of going to one of those Alzheimer’s cafés, but then I also think: what am I going to do there. You have to solve it yourself. You have to solve it by yourself, others can’t do very much for you.”*

Several participants had a negative outlook on the future which was often shaped by images of advanced dementia. They thought that quality of life would decline as a result of losing abilities. *P04: “So I think, if I get worse and it gets so bad that, well, I can’t do certain things and I don’t recognize (loved one’s name) for example, yes, then of course I’m done.”*

Some participants mentioned having difficulty accepting their mortality. Finally, they were afraid that the disease would tarnish the image of their “old self.” *C09: “He just doesn’t want to lose the image the kids have of him. Of course he doesn’t want to lose anything, it’s as simple as that, I wouldn’t want that either if I were him.”*

Other participants viewed the inevitable decline more with greater acceptance. They realized that a cure was not possible and were aware of the terminal nature of dementia. *P08: “You know it’s happening, so you can’t say, it'll be better. Again and again another little stone falls off. Walking to your death, but you also have to be pragmatic about it. You know it’s happening, you can see it happening. You start to do as many of the little things you can still do.”*

### Orientation toward the future versus focus on the present

Participants showed contrasting attitudes toward the future including advance care planning. Some focused on the present while others had wishes in advance that would necessitate advance care planning. They anticipated reaching agreements in the future, such as do-not-resuscitate orders. Quality of life was mentioned as a factor to take into account when making treatment decisions. *C06: (talking about drawing up the advance euthanasia directive) “You (wife) are very quick to do that and now you’re already working on ‘under what conditions do I want to live and under what conditions do I not.’ (. . .) It has nothing to do with ageing, but she is constantly talking about quality of life and doesn’t always link that to age.”* Many participants with dementia stated suffering should be avoided in the final phase and that they would not always want their life prolonged. *P05: “I could imagine a situation arising in which I think: enough. Then it’s also a self-chosen end.”* Most people with dementia preferred not to go to a nursing home, with two stating they would have no desire to live if admission were necessary. Euthanasia, which was brought up by the dyads themselves, would (six cases) or would not (two cases) be an option in case of further decline.

Multiple participants wished for certainty toward the future. They considered reliable information about the prognosis important. Some dyads were already thinking about future scenarios and making preparations by arranging care at home or visiting day-care facilities. Participants with dementia wanted to make arrangements in advance while they were still capable of doing so. These arrangements could reassure dyads. *P09: “On the one hand, as strange as it may sound, it can give you peace of mind. (. . .) Because things have been arranged; that you can think about it while you were still healthy in body and soul.”*

By contrast, other participants highlighted the uncertainty of the future due to the disease’s unpredictability. Therefore, they felt unable to asess in advance what support would be needed. *C08: “Things will comes as they come and I can plan today for the situation a year from now, but maybe it won’t be like that at all. I can’t plan for it.”* Moreover, some dyads stated that the wishes of the person with dementia might change over time. In particular, they expected the response to the decline to become more accepting. *P04: “Because look, this roadmap is probably, of course it’s small steps every time so then I think well, that’s not so bad after all, that’s not so bad.”* Some participants doubted the usefulness of future planning as they believed that advance directives, especially euthanasia, could not be granted without capacity to confirm their wish.

Several dyads chose to focus on the present by adopting a day-to-day attitude. Many found thinking of the future stressful and confronting. *C09: “Interviewer: Why don’t you think about that (the future) just yet? Because it is very confronting. (. . .) Then I find it very confronting to think about it.”*

Caregivers more frequently than people with dementia preferred to make decisions in the future rather than in advance. They planned to leave the situation unchanged until action was necessary, such as after incidents. Dyads had confidence in caregivers as acting legal representative, capable of making appropriate decisions. *C02: “But I’m quite a decisive type of person. And I think I can switch gears quickly when necessary. I don’t need to have a plan for that, I very quickly make that plan.”*

## Discussion

### Main findings

A sense of self and connection to others were essential components of quality of life in our study on young-onset dementia, both in the present and future. Participants’ perspectives on the disease and its implications varied greatly, as did their coping styles, which ranged from acceptance to resistance. Expectations of a decrease in quality of life could adversely affect the outlook on the future and thus reduce the willingness to engage in advance care planning. For those who did engage, quality of life was explicitly considered when making treatment decisions.

### What this study adds

Our study is the first to define the core elements of quality of life in the context of palliative care and advance care planning for people with young-onset dementia. Dementia research also found quality of life domains including retaining identity, feeling useful, maintaining independence, feelings of attachment, and social interaction.^31,32^

The interviews showed that participants feel stigmatized, making them less willing to talk openly about their disease with others. They live with existential questions, which makes acceptance difficult. People with young-onset dementia have to deal with a serious disruption of the life-cycle that aggravates the negative experience of receiving their diagnosis.^33,34^ However, we also identified positive ways of coping, such as acceptance, adaptability, and openness to support. These can help people with young-onset dementia maintain personal agency.^35^ A study into early-stage Alzheimer’s disease highlighted that responses and coping styles differ between individuals regardless of age of onset.^36^ Therefore, living with dementia is first and foremost an individual experience.^37^

Quality of life, based on personal judgement, was found to play a crucial role in making treatment decisions, which is line with a palliative care approach. Our study confirmed the relevance of the main palliative care goals as identified in an international Delphi study.^10^ First, the significance of maintaining a sense of self supports the care goal of protecting identity and respecting personhood, thereby affirming the palliative care domain of person-centered care. Second, the goal of maintaining control over function, which contributes to essential quality of life aspects such as connection, autonomy, and engagement in activities, was also reflected in our findings. Third, a focus on comfort instead of life prolongation, is consistent with research on mostly older people with dementia^38,39^ and nursing home residents with more advanced young-onset dementia.^40^ Contrary to previous hypotheses, family caregivers in our study did not prefer life-prolonging treatment.^13^ Finally, the well-being of family caregivers was also found to be important, underscoring the need of palliative care to address their needs.

The relatively high number of advance directives in our small sample as compared to the general population is in line with a previously suggested active role of people with young-onset dementia in advance care planning.^13,41^ Both persons with dementia and family caregivers stated that the preferences of the person with dementia should be decisive, even if the family caregiver had a different opinion. These findings show advance care planning as a process to protect autonomy in which family plays a vital role.^20^ By contrast, previous dementia research indicated that caregivers may struggle whether to adhere to the established preferences in advance-care planning.^39,42^

Reasons for not engaging in advance care planning included the unpredictable nature of dementia and adopting a day-to-day attitude, as found in Flemish persons with young-onset dementia.^19^ Moreover, in our study, some caregivers felt no urgency to engage in advance care planning and felt confident they would be able to make decisions as needed. Finally, participating in advance care planning could be confronting, a well-known theme among people with dementia.^19,39,42^ Fears of compromised quality of life, for example by increased dependency, contributed to this confronting nature, thereby preventing people from engaging in advance care planning. Negative images of advanced dementia played a significant role in shaping views on the future and were, as a result, linked to considerations of euthanasia. This finding corresponds with previous studies of euthanasia in dementia.^43,44^ However, these images may not reflect how people will actually perceive their situation in the future.^42^

### Strengths and limitations

We interviewed individuals with young-onset dementia and their caregivers separately, which minimized interference and allowed for addressing both perspectives. The in-depth interviews yielded rich data. Iterative adjustments to the interview guide facilitated the exploration of new insights. The sample exhibited heterogeneity in terms of gender, age, dementia type, and disease duration. However, perspectives of children as family caregivers or people with different cultural backgrounds are not represented, which limits transferability. Cultural background is particularly relevant, as cross-cultural differences have been found regarding perpections of good end-of-life care and the acceptance of ACP.^14,16,38^ We did not explore the viewpoints of individuals without close family caregivers or those with severed family ties. The selection of harmonious couples who agreed to participate may have excluded individuals with young-onset dementia who are opposed to family involvement or a relationship-centered approach. Additionally, recruitment was conducted through case managers who might have favored dyads with stronger relationships or with a particular interest in advance care planning.

### Implications

It is crucial for professionals to recognize the individualized nature of living with young-onset dementia. Not all people with young-onset dementia and family caregivers approach conversations about future treatment and care proactively despite an emphasis on autonomy. This underscores the need for professionals to take the initiative. While dyads often placed significant trust in family caregivers to make future decisions, their perspective may differ from that of the person with dementia. Therefore, conversations about future care should also be conducted separately with the person with dementia and their family caregiver, allowing each to express their perspectives before aligning them. Sense of self and connection to others can serve as starting points for advance care planning conversations including discussing palliative care. Understanding individuals’ views toward the future, particularly in terms of their perceived quality of life, may help remove barriers to engaging in advance care planning. By framing future care discussions as a means to protect their future sense of self, healthcare professionals may help patients feel empowered to make decisions. Future studies could include a more diverse sample in terms of family context and cultural background. Patient and Public Involvement could improve methods, including recruitment strategies, and help align research questions more closely with the priorities and experiences of people with young-onset dementia themselves. Additionally, it would be interesting to follow people in longitudinal studies to examine whether the perceptions of quality of life change over the course of the disease as the participants in our study anticipated.

## Conclusions

Perspectives on young-onset dementia and its impact vary among people with young-onset dementia and their family caregivers. Preserving a sense of self and connection to others is crucial for maintaining quality of life. These topics should be discussed in advance care planning, as they can help establish relevant palliative care goals.

## Supplemental Material

sj-docx-1-pmj-10.1177_02692163251324796 – Supplemental material for Advance care planning and quality of life: A qualitative interview study in people with young-onset dementia and their family caregivers Supplemental material, sj-docx-1-pmj-10.1177_02692163251324796 for Advance care planning and quality of life: A qualitative interview study in people with young-onset dementia and their family caregivers by Jasper Maters, Marieke Perry, Ton de Wit, Raymond T.C.M. Koopmans, Marjolein E. de Vugt, Christian Bakker and Jenny T. van der Steen in Palliative Medicine
