# Supplementary material for: Advance care planning and quality of life: A qualitative interview study in people with young-onset dementia and their family caregivers
Source: Palliat Med. 2025 Mar 15;39(5):563–73. doi: 10.1177/02692163251324796 (PMC12033381; doi:10.1177/02692163251324796)
Supplement: sj-docx-1-pmj-10.1177_02692163251324796 – Supplemental material for Advance care planning and quality of life: A qualitative interview study in people with young-onset dementia and their family caregivers [file sj-docx-1-pmj-10.1177_02692163251324796.docx]

**Supplement S1 Interview guide**

| Person with dementia | |
| --- | --- |
| 1. Elicitation technique based on picture/photograph/item that the participants brought with them | |
| *Can you talk a little bit more about that?*  *What does that mean to you?* | |
| 1. Quality of life | |
| *What do you enjoy (doing)?*  *What do you find valuable in life?* | |
| 1. Care and young-onset dementia | |
| Primary question  *What is it like for you to have dementia?* | Possible follow-up questions  *Are there things you can no longer do because of your illness?*  *Do you experience support from people around you?*  *What is it like for you to receive help from professional caregivers?* |
| 1. Future | |
| Primary questions  *Do you ever think about the future?*  *Do you think the dementia will get worse?* -Increasing memory loss  -Increased dependency  -Relocation due to increased dependency  Additional question after fifth dyad interview  *What would you like to keep for the rest of your life? What not?* | Prompt question  If yes: *What do you think about specifically?*  If no: *Can you explain why you don't, or would rather not think about the future?*  If yes: *What is it like for you to know that the dementia is slowly getting worse?*  If no: *Can you tell us how you think the dementia will progress?* |
| 1. Advance care planning | |
| Original question  *Do you ever think about the last stage of your life?*  Adjusted question after fifth dyad interview  *Do you ever think about the late stages of life and/or the last stage of life (end of life)?* | Possible follow-up questions *Do you have any particular wishes?* -Role of loved ones/informal caregiver(s) -In case of certain symptoms -Symptom relief -At a particular location -Care by caregivers -Certain Rituals  -Euthanasia [do not introduce this yourself,  **only** if respondent brings it up]  *Have you talked to anyone about this? If so, with whom?*  -GP (or other physician, or case manager or district nurse) -Loved ones/informal caregiver(s)  *What do you think is the best way to provide the care you need in the future?* |
| 1. *Tips for healthcare professional (added after fifth dyad interview)* | |
| *Do you have any tips for caregivers on how to discuss future care?* | |
| Family caregiver | |
| 1. Elicitation technique based on picture/photograph/item the participants brought with them | |
| Similar to questions for person with dementia, from the other person's perspective | |
| 1. Quality of life | |
| Primary questions  *What is important in life for your loved one?*  *What makes life worth living for your loved one?*  *What is important for your loved one to have good quality of life?*  *What does quality of life mean to you? What makes life worth living for you?* | |
| 1. Care and young-onset dementia | |
| Similar to questions for person with dementia, from the other person's perspective | |
| 1. Future: | |
| Similar to questions for person with dementia, from the other person's perspective  Additional question  *What would you like to keep for the rest of the life of your loved one? What not?* | |
| 1. Advance care planning | |
| Similar to questions for person with dementia, from the other person's perspective  Additional question  *What do you think is the best way to provide the care your loved one will need in the future?* | |
